# Broadband Circular Polarizer Based on Chirped Double-Helix Chiral Fiber Grating

**DOI:** 10.3390/ma15093366

**Published:** 2022-05-07

**Authors:** Linlin Xue, Bras Samuel Malumba Timoteo, Weiwei Qiu, Zhongpeng Wang

**Affiliations:** School of Information and Electronic Engineering, Zhejiang University of Science and Technology, Hangzhou 310023, China; samuelbras24@hotmail.com (B.S.M.T.); weiweiqiu2015@163.com (W.Q.); wzp1966@163.com (Z.W.)

**Keywords:** chiral fiber grating, leaky mode coupling, circular polarizer, broadband

## Abstract

We propose an all-fiber broadband circular polarizer based on leaky mode coupling and a phase-matched turning point (PMTP) in a chirped, double-helix, chiral, long-period, fiber grating (CLPG). The CLPG was coated with a material in which the refractive index was higher than that of the fiber cladding, enabling the coupling of the core mode to leaky modes to achieve a desired extinction ratio. The complex coupled-mode theory was employed to investigate the coupling mechanism and conditions under which the desired coupling efficiency could be achieved. Moreover, the PMTP in phase-matched curves, which resolved the conflict between the operating bandwidth and the grating pitch range of the CLPG and made a large bandwidth with a small grating pitch possible, was used in the design to achieve a compact structure. Finally, two broadband circular polarizers with an extinction ratio above 25 dB were simulated; one had a bandwidth of over 120 nm and a length of 3.5 cm, and the other had a bandwidth of over 300 nm and a length of 8 cm.

## 1. Introduction

Circular polarization technology has important applications in the fields of optical fiber sensing and communication [1,2,3,4]. Circular polarizers, which are usually formed from bulk elements (e.g., linear polarizers, waveplates and polarization rotators), are key devices in circular polarization technology, but the devices realized according to this scheme are bulky and may reduce the stability of the whole system. Furthermore, chiral structure metamaterials have also been employed to manipulate the polarization of light and corresponding circularly polarized devices were demonstrated, such as broadband circular polarizers, linear-to-circular polarization converters and polarization rotators [5,6,7,8]. However, for applications in optical fiber sensing and communication, all-fiber-based circular polarizers are more desirable.

In 2004, Kopp proposed double-helix, chiral, long-period, fiber gratings (CLPG) fabricated by twisting high birefringence (Hi-Bi) fibers with pitches of hundreds of micrometers [9] and experimentally demonstrated that, besides the function of band-rejection like that of the conventional long-period fiber gratings, this kind of fiber grating had a remarkable property of polarization-selective coupling of circularly polarized modes [9]. Subsequently, a series of theoretical and experimental investigations of CLPG were reported [10,11,12,13,14,15,16], and multiple applications were demonstrated [17,18,19,20,21,22,23,24,25,26,27,28,29], such as all-fiber circular polarizers, orbital angular momentum mode converters, band-rejection filters, torsion sensors and current sensors.

The property of polarization-selective coupling of circular polarized modes made the chiral fiber grating suitable to be used as an all-fiber circular polarizer, and circularly polarized devices based on various chiral fiber gratings were reported and demonstrated. In [9,10,11,17,18], all-fiber circular polarizers created by twisting Hi-Bi fibers were demonstrated; in [30,31,32], all-fiber circular polarizers and circular polarization beam splitters based on twisted photonic crystal fibers were reported; and in [33], circular polarization filters based on two consecutively twisted single-mode fibers with opposite helicities were demonstrated. In this paper, we focus on the circular polarizers formed by twisting Hi-Bi fibers, which are commercially available and have low connection loss with standard single-mode fibers. 

The property of polarization-selective coupling of circular polarized modes in CLPG is explained using coupled-mode analysis [12,13,14,15], which can be briefly expressed as follows: a CLPG has different propagation constants for its right- and left-handed circularly polarized modes. For a CLPG with a twist pitch of hundreds of micrometers and a given twist handedness (right- or left-handed), the co-handed (right or left) circularly polarized core mode phase matches and couples to the co-propagating cross-handed (left or right) circularly polarized cladding modes at certain resonant wavelengths. At these wavelengths, the co-handed (right or left) circularly polarized core mode suffers from loss, while the cross-handed circularly polarized core mode transparently passes through the CLPG. Using one of these resonant couplings, a cross-handed circular polarizer can thus be developed. 

However, the bandwidth of this resonant-coupling-based polarizer spans a range of only tens of nanometers [9,10,11], which is too narrow a range for most applications. Kopp et al. demonstrated a broadband, circular polarizer by decreasing the twist pitch to tens of micrometers and by exploiting the coupling of core mode with radiation modes [9]. Nevertheless, this scheme may increase the difficulty of implementation due to the shorter twist pitch control. Inspired by the adiabatic coupling principle, which has been successfully employed in microwave physics to achieve broadband couplers, we previously proposed an adiabatic, broadband, circular polarizer based on a double-helix CLPG with a slowly varying twist rate (a chirped CLPG) [17]. However, to fulfill the adiabatic condition, the length of the circular polarizer must be sufficiently long (with a length of 41.5 cm for a 60 nm bandwidth in [17]). Although multi-mode phase-matching along the grating and coupling of core mode with a lossy mode were used to relax the adiabatic condition, a length of 20 cm was still required to achieve the desired extinction ratio [17]. To overcome this problem, we proposed a chirped CLPG coated with a high refractive index (RI) material for coupling of core mode with leaky modes [18]. Due to leakage, the power that couples from the core mode to leaky modes radiates out of the fiber and cannot be recoupled back to the core mode, which would otherwise be desirable to achieve a high extinction ratio with a short coupling length. Using a coated chirped CLPG, a circular polarizer with a bandwidth of 60 nm and a length of 9 cm was demonstrated [18]. However, to achieve a broader bandwidth, a correspondingly larger grating pitch range as well as a longer grating length, which is not beneficial for practical applications, are required. In this study, we introduce a phase-matching turning point (PMTP) in the design, which has been successfully used in increasing fiber sensitivity and in the bandwidth broadening of fiber filtering [34,35,36]; we propose a scheme that efficiently combines leaky mode coupling and PMTP. This scheme retains the advantages of leaky mode coupling while making use of PMTP to resolve the conflict between the operating bandwidth and grating pitch range, demonstrating a circular polarizer with broad bandwidth and a compact structure. 

In Section 2, the theoretical model of the coated double-helix CLPG is described using complex, coupled-mode theory. The coupling mechanism and PMTP properties are investigated on this basis. In Section 3, simulations are carried out based on the theoretical analysis, and two broadband circular polarizers with different bandwidths are demonstrated for various applications. Finally, conclusions are drawn in the last section. 

## 2. Theoretical Analysis 

### 2.1. Coated Uniform CLPGs

**Figure 1:**
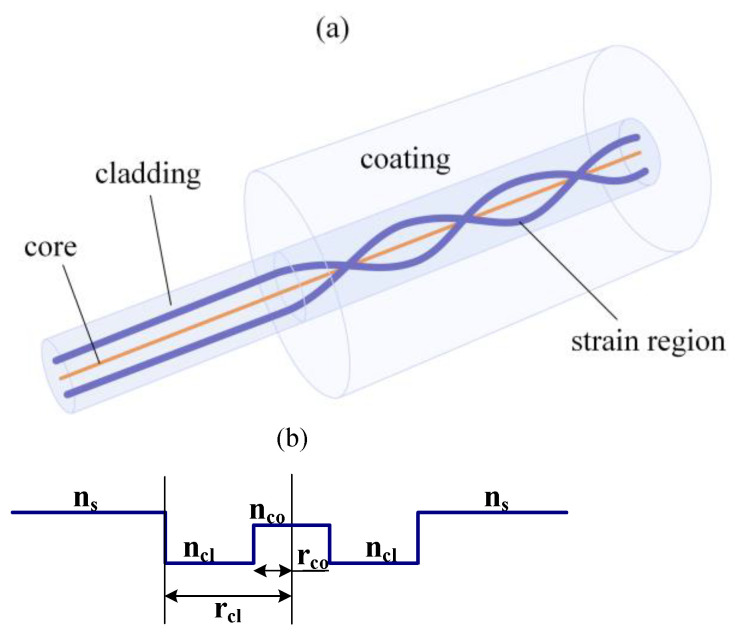
Theoretical model of coated CLPG. (**a**) Sketch of CLPG formed by twisting a panda fiber and coated with a high-RI material; (**b**) index profile of CLPG.

Figure 1a shows a theoretical model of a coated uniform CLPG formed by twisting a Hi-Bi fiber with right-handed rotation and a constant twisting rate. Here, a panda-type, polarization-maintaining fiber with index-matched cladding is used, and the twisted part is coated with a material in which the RI is higher than that of the cladding. For the materials used for coating, polymers, which possess high RI and good mechanical properties, would be a good choice [37,38], and are convenient when combined with or coated over optical fibers. 

Figure 1b shows the corresponding RI profile of a perfect untwisted isotropic fiber, which is considered as a reference fiber. Here, n_co_ and n_cl_, and r_co_ and r_cl_ are the RIs and radii of the core and cladding, respectively. The thickness of the coating is assumed to be large enough for the effects of air on the fiber modes to be neglected. Because the coating RI is higher than that of the cladding, the cladding modes of the reference fiber do not experience total internal reflection and thus turn into leaky modes [39]. For practical fabrication, a sufficiently thick polymer coating should be used, i.e., 10 mm, to minimize the effect of air on the fiber modes. Hence, besides forming a leakage structure, the polymer coating also acts as a jacket for the Hi-Bi fiber and increases the mechanical property of the CLPG. 

In our simulation, the guided core mode and leaky modes in the coated reference fiber, which is referred to as a leaky or hollow dielectric waveguide, were calculated by using an equivalent model terminated by a perfect electric conductor-backed perfectly matched layer [40]. 

To analyze the coated CLPG, we used complex coupled-mode theory, which was derived using a local mode approach [13,17,18]. For a CLPG with a right-handed twisted structure, the right-handed circularly polarized core mode (RCPCM) couples with a co-propagating left-handed circularly polarized leaky mode (LCPLM); the complex coupled-mode equation describing this coupling can be expressed as follows [13,17,18]:

(1) $$\frac{d}{dz}\left[\begin{array}{l} W_{\mathrm{co}}^{r}\\ W_{n}^{l} \end{array}\right]=\left[\begin{array}{cc} -j(\beta_{\mathrm{co}}-\tau ) & C\\ -C & -j(\beta_{n}+\tau ) \end{array}\right]\left[\begin{array}{l} W_{\mathrm{co}}^{r}\\ W_{n}^{l} \end{array}\right],$$

where *W_co_^r^* and *W_cl_^l^*, and *β*_co_−*τ* and *β*_n_+*τ* are the amplitudes and phase constants of the RCPCM and the LCPLM in the CLPG, respectively. *β*_co_ and *β*_n_ are the phase constants of the core mode and leaky mode in the reference fiber, respectively; notably, *β*_co_ is a real number, whereas *β*_n_ is a complex number with a real part *β*_n_^r^ and an imaginary part–*β*_n_^i^. *τ* is the twist rate of the chiral fiber grating and is positive for right-handed rotation and negative for left-handed rotation; *C* represents the coupling coefficient, which is defined as follows:

(2) $$C=\frac{\omega \varepsilon_{0}}{2}\iint \left(\Delta \varepsilon_{x}-\Delta \varepsilon_{y}\right)\;e_{01}\cdot \;e_{0n}dS,$$

where Δ*ε_x_* and Δ*ε_y_* are the anisotropic perturbations for x- and y- polarized light to the dielectric constant distribution in the cross section of the reference fiber, induced by the birefringence. **e**_01_ and **e**_0n_ are the normalized modal fields of the core mode and the leaky mode in the reference fiber, respectively. Because of coupling of core mode with leaky modes, the coupling coefficient *C* is complex, but its imaginary part is ignored in the following analysis and calculations because of its small magnitude and negligible contributions to the coupling process.

Equation (1) can be simplified by retaining only terms that involve the amplitudes of the two modes:

(3) $$\frac{d}{dz}\left[\begin{array}{c} A_{co}\\ A_{n} \end{array}\right]=\left[\begin{array}{cc} -j\delta & C\\ -C & j\delta -\beta_{n}^{i} \end{array}\right]\left[\begin{array}{c} A_{co}\\ A_{n} \end{array}\right],$$

where the novel amplitudes are $A_{co}=W_{co}^{r}\exp(j\frac{\beta_{co}+\beta_{n}^{r}}{2}z)$ and $A_{n}=W_{n}^{l}\exp(j\frac{\beta_{co}+\beta_{n}^{r}}{2}z)$; $\delta =\frac{\beta_{co}-\beta_{n}^{r}-2\tau }{2}$ is the phase detuning factor, which is the real part of the phase constant difference between the RCPCM and the LCPLM in the CLPG. Furthermore, *δ* = 0 is defined as the phase-matching condition for this complex mode coupling, under which the coupling between the RCPCM and LCPLM is strongest.

To obtain the analytical solution, Equation (3) is transformed into a second-order differential equation only on *A*_co_ by eliminating *A*_n_, as shown in Equation (4). Here, the initial conditions *A_co_* = 1 and *A_n_* = 0 at the input end *z* = 0 were used.

(4) $$\{\begin{array}{c} \frac{d^{2}A_{\mathrm{co}}}{dz^{2}}+\beta_{n}^{i}\frac{dA_{\mathrm{co}}}{dz}+(C^{2}+\delta^{2}+j\delta \beta_{n}^{i})A_{\mathrm{co}}=0\\ A_{\mathrm{co}}(0)=1,\frac{dA_{\mathrm{co}}}{dz}{|}_{z=0}=0 \end{array}$$

When the phase-matching condition *δ* = 0 is fulfilled, Equation (4) has a form similar to that of a damped-oscillation equation. In addition, we define a loss factor $p=\frac{-\beta_{n}^{i}}{2C}$, based on which the analytical solutions of Equation (4) under the phase-matching condition can be easily obtained and explicitly categorized into three cases:

When −1 < *p* < 0,

(5) $$\{\begin{array}{c} A_{co}=\exp(-\frac{\beta_{n}^{i}}{2}z)\left[\cos(\sqrt{1-p^{2}}Cz)-\frac{p}{\sqrt{1-p^{2}}}\sin(\sqrt{1-p^{2}}Cz)\right]\\ A_{n}=-\exp(-\frac{\beta_{n}^{i}}{2}z)\frac{1}{\sqrt{1-p^{2}}}\sin(\sqrt{1-p^{2}}Cz) \end{array}$$

when *p* = −1,

(6) $$\{\begin{array}{c} A_{co}=(1+Cz)\exp(-\frac{\beta_{n}^{i}}{2}z)\\ A_{n}=-Cz\exp(-\frac{\beta_{n}^{i}}{2}z) \end{array}$$

when *p* < −1,

(7) $$\{\begin{array}{c} A_{co}=\exp(-\frac{\beta_{n}^{i}}{2}z)\left[\cosh(\sqrt{p^{2}-1}Cz)-\frac{p}{\sqrt{p^{2}-1}}\sinh(\sqrt{p^{2}-1}Cz)\right]\\ A_{n}=-\exp(-\frac{\beta_{n}^{i}}{2}z)\frac{1}{\sqrt{p^{2}-1}}\sinh(\sqrt{p^{2}-1}Cz) \end{array}$$

**Figure 2:**
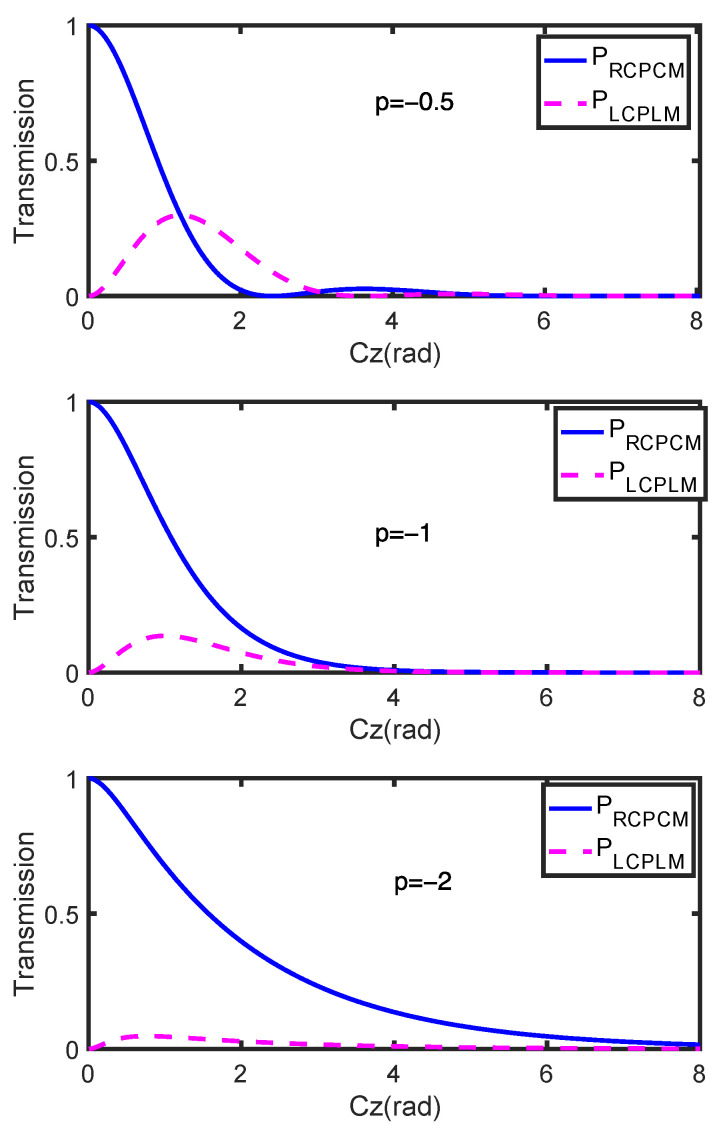
Transmission of the RCPCM and LCPLM with different loss factors *p*.

Based on Equations (5)–(7), the transmission of the RCPCM and LCPLM is simulated for the three cases, as shown in Figure 2. The power evolution of the RCPCM resulting from coupling can be deduced from Figure 2 and is similar in form to that of damped oscillations. When −1 < *p* < 0, the transmission of the RCPCM along the coupling length is periodic with exponential attenuation, which corresponds to the underdamping state of damped oscillations; when *p* = −1, the transmission of the RCPCM along the coupling length is attenuated monotonously and almost exponentially, which corresponds to the critical damping state of damped oscillations; and when *p* < −1, the transmission of the RCPCM is similar to the case for *p* = −1, but with a smaller attenuation rate, which corresponds to the overdamping state of damped oscillations. 

**Figure 3:**
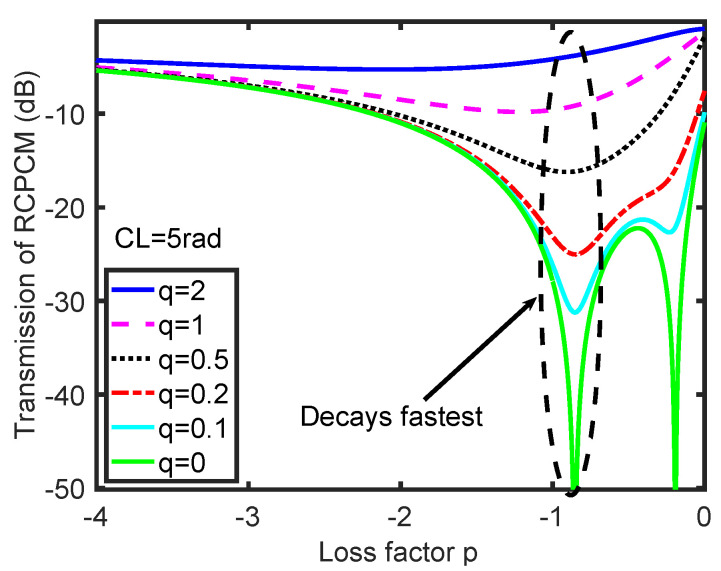
Variation in the transmission of the RCPCM with loss factor *p* for different detuning factors *q*.

**Figure 4:**
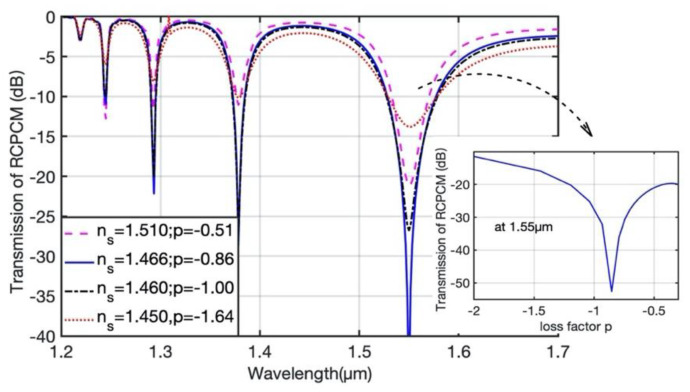
Transmission of the RCPCM with different-RI coatings.

However, the analytical solutions, Equations (5)–(7), and the corresponding transmission simulations are obtained under the phase-matching condition, which can only be fulfilled at certain wavelengths. To study the coupling process when the phase-matching condition is not fulfilled, we define a detuning factor q=δ/2C and simulate the transmission of the RCPCM with a loss factor *p* for different detuning factors *q*, as shown in Figure 3. The highest coupling efficiency occurs at *q* = 0, where the phase-matching condition is strictly fulfilled; when *q* increases, indicating that the coupling gradually deviates from phase-matching condition, the coupling efficiency decreases. The variation of the transmission of RCPCM with *p* is not as explicit as that with *q*, but the variation trend coincides with the analytical solutions in Equations (5)–(7). Overall, the transmission of the RCPCM decays fastest when the loss factor *p* is approximately −0.9 for various *q*, as indicated by the oval in Figure 3. Hence, to achieve the desired coupling efficiency within a short coupling length, the loss factor *p* must be set around −0.9. To further validate this point, we simulated the transmission spectra of a coated uniform CLPG with different loss factors *p*, as shown in Figure 4.

The parameters used in the simulation and the remainder of this study are based on the commercial, polarization-maintaining fiber PM1300-XP provided by THORLABS. The fiber’s core and cladding radii are 4 and 125 μm, respectively; its numerical aperture is 0.12, its beat length is 4 mm, and its operating wavelength range is 1270 to 1625 nm. The twist pitch Λ, which is defined as Λ=2π/τ, is 880 μm to enable the phase-matching condition between RCPCM HE_1,1,_ and LCPLM HE_1,6_ to be fulfilled at 1.55 μm, and the length of the CLPG is 4 cm. The effects of dispersion are also addressed using the Sellmeier function [41] in the simulation. Figure 4 shows that the transmission spectra of RCPCM are similar to those of long-period fiber gratings in high-RI surroundings [39], whereas the coupling in the CLPG is polarization selective. Furthermore, the transmission of the RCPCM varies with the loss factor *p*, and the variation is in accordance with the three states indicated by the analytical solutions. However, Figure 3 only yields the transmission loss of the RCPCM with several discrete values of *p*; hence, we simulated the transmission of the RCPCM with continuous *p* at one of the resonant wavelengths, as shown in the inset of Figure 4. For values of *p* around −0.9, the coupling efficiency is highest, providing a high extinction ratio with a short grating length. Nevertheless, in a uniform CLPG, strong coupling only occurs at certain wavelengths where the phase-matching condition is fulfilled. This yields a circular polarizer with a bandwidth of only tens of nanometers, which is too narrow for most applications. To obtain a broadband circular polarizer, we used a chirped coated CLPG with a varying twist rate that enabled the phase-matching condition to be fulfilled for a large range of wavelengths. 

### 2.2. Coated Chirped CLPGs

A chirped CLPG has a varying twist rate *τ*(z) as well as a variable grating pitch Λ(z), which marks the main difference from a uniform one. To describe the rate of change in pitch Λ(z) along the grating length, we define the chirp coefficient *F* as ΔΛ/L, where ΔΛ is the difference between the maximum and minimum pitches of the chirped CLPG. To achieve a high extinction ratio for a given grating length, the chirped coefficient *F* as well as ΔΛ must be small. This is because, for a high chirped coefficient, the grating pitch changes rapidly along the CLPG, and the effective coupling length for every pitch is short, yielding a low coupling efficiency and low extinction ratio.

The coupled-mode equation for a coated chirped CLPG has the same form as Equation (1) but with a varying twist rate *τ*(z). Due to the varying twist rate, obtaining analytical solutions is more difficult than in the case of a coated uniform CLPG. Here, the transfer matrix method was used in the analysis and simulation of the coated chirped CLPG, where the chirped CLPG was divided into *M* sections, and each section was regarded as a uniform one. Therefore, the coupling properties between the RCPCM and LCPLM for each section were the same as those of a uniform CLPG, and the overall transmission of the chirped CLPG could be considered a superposition of the *M* sections of the uniform CLPGs. 

The phase-matching condition for the coated chirped CLPG is expressed as Equation (8), which can be fulfilled in a certain wavelength range:

(8) $$\delta =\frac{\beta_{co}-\beta_{n}^{r}-2\tau (z)}{2}$$

**Figure 5:**
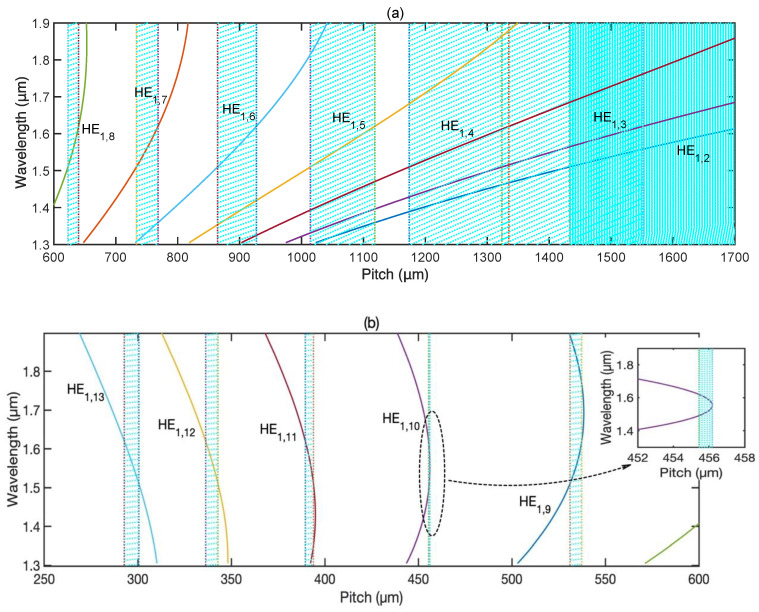
Phase-matching curves of a coated chirped CLPG for an operating wavelength range from 1.50 to 1.62 μm. (**a**) Phase-matching curves for grating pitch range from 600 to 1700 μm, where lower-order LCPLMs from HE_1,2_ to HE_1,8_ have the chance to phase match with the RCPCM. (**b**) Phase-matching curves for grating pitch range from 250 to 600 μm, where higher-order LCPLMs from HE_1,9_ to HE_1,13_ have the chance to phase match with the RCPCM.

Based on Equation (8), phase-matching curves (PMCs) for the RCPCM and different-order LCPLMs were calculated, as shown in Figure 5, which clearly reveals the relationship between the resonant wavelength and grating pitch. Figure 5a corresponds to the grating pitch range from 600 to 1700 μm, where lower-order LCPLMs, i.e., from HE_1,2_ to HE_1,8_, have the chance to phase match with the RCPCM for the optical wavelength of communication, while Figure 5b corresponds to the grating pitch range from 250 to 600 μm, where higher order LCPLMs, i.e., HE_1,9_ to HE_1,13_ have the chance to phase match with the RCPCM for the optical wavelength of communication.

Using a chirped CLPG with an operating wavelength range from 1.50 to 1.62 μm as an example, the corresponding pitch ranges satisfying the phase-matched condition for different-order LCPLMs are illustrated by the shaded parts in Figure 5. The pitch range for low-order modes is larger than for high-order modes, which creates a high chirped coefficient and hinders the production of a high extinction ratio with a short grating length. Furthermore, the pitch ranges for low-order modes overlap with their neighboring ones, indicating that numerous modes must be taken into consideration, thus complicating the analysis and design of a broadband circular polarizer. Consequently, high-order modes are preferrable in the design of a broadband circular polarizer that is based on a coated chirped CLPG, especially the modes that have a PMTP within the operating wavelength range, such as HE_1,10_. To clearly show the PMTP of HE_1,10_, we zoom in on the PMC of HE_1,10_, as shown in the inset of Figure 5b. In this case, the pitch at the PMTP can be set as the maximum pitch of the chirped CLPG, and the minimum pitch can be determined by the bandwidth of the chirped CLPG. When the grating pitch decreases from the PMTP, the band of the chirped CLPG broadens both to longer and shorter wavelengths. Consequently, the pitch range required for a broadband circular polarizer based on the chirped CLPG decreases dramatically by taking advantage of the PMTP. Furthermore, for this pitch range, only one LCPLM can fulfill the phase-matching condition with the RCPCM and must be considered in the coupled-mode equation, rendering the analysis simple and intuitive.

## 3. Design and Simulations

We focused on the design of two broadband circular polarizers with operating wavelength ranges from 1.50 to 1.62 μm and from 1.30 to 1.62 μm. The design procedure is as follows: first, select an LCPCM which has a minimum pitch range within the operating wavelength range, to couple with RCPCM. Second, ensure that the loss factor is approximately −0.9 by choosing an appropriate coating RI. Third, determine the grating length using the given extinction ratio. 

**Figure 6:**
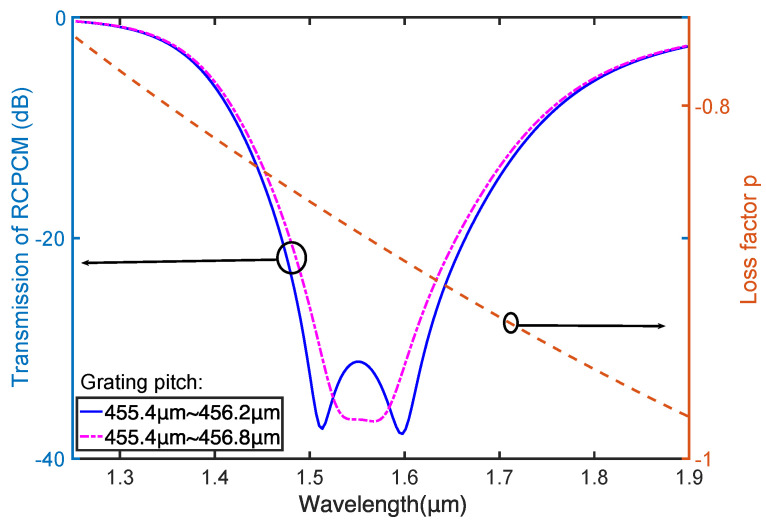
Transmission of the RCPCM and corresponding loss factor in coated chirped CLPG with a bandwidth of 100 nm.

For an operating wavelength range from 1.50 to 1.62 μm, the LCPLM selected to couple with the RCPCM is HE_1,10_, and its corresponding pitch range is from 455.4 to 456.2 μm, which is the minimum of all the pitch ranges, as shown in Figure 5b. The coating RI is chosen to be 1.65, which yields a loss factor of approximately −0.9 within the operating wavelength range. The grating length is set to be 3.5 cm to make the transmission loss of the RCPCM greater than 25 dB within the operating wavelength range, which is sufficient for most applications [9]. The transmission of the RCPCM and the corresponding loss factor of the chirped CLPG were simulated with these parameters, shown by the solid and dashed lines, respectively, in Figure 6. The bandwidth coincides with that designed using the PMCs in Figure 5, and the extinction ratio within the bandwidth is larger than 25 dB. Owing to the remarkable characteristics of leaky mode coupling and PMTP, a circular polarizer with a bandwidth of 120 nm and a length of only 3.5 cm was achieved, with significantly improved performance compared with that obtained in Refs. [17,18].

Nevertheless, a peak appears at the center of the transmission curve, resulting from the low coupling efficiency at the PMTP. Because the maximum grating pitch is set just at the PMTP in our design, the efficient coupling length between the RCPCM and LCPLM at the center wavelength is short compared with other wavelengths; this leads to low coupling efficiency as well as a peak in the transmission curve. Hence, the peak can be eliminated by slightly expanding the maximum pitch and by increasing the efficient coupling length at the PMTP. As indicated by the dash-dotted line in Figure 6, when the maximum grating pitch is expanded to 456.8 μm, the peak at the center disappears, giving a flat transmission band. 

**Figure 7:**
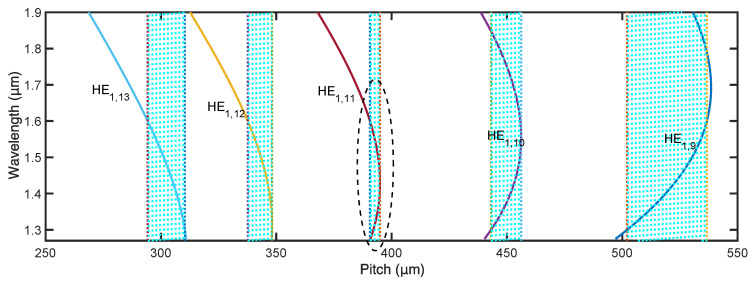
Phase-matching curves of coated chirped CLPG for operating wavelength range from 1.30 to 1.62 μm.

**Figure 8:**
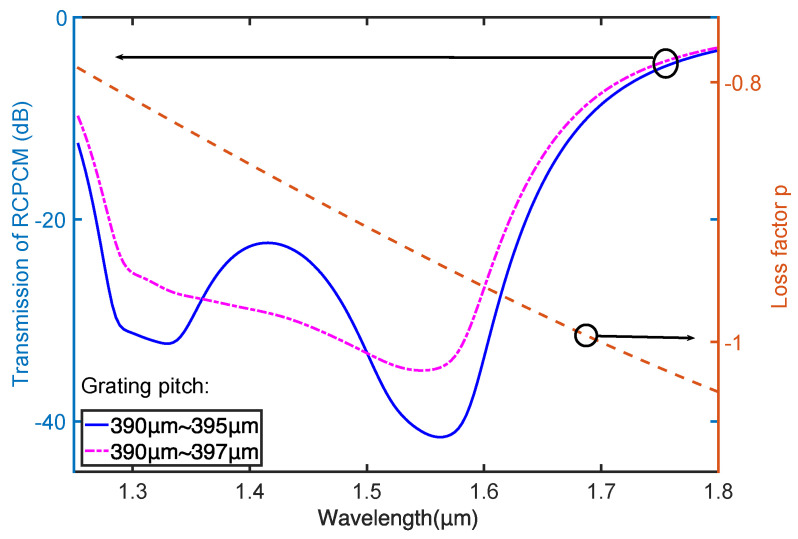
Transmission of RCPCM and corresponding loss factor in coated chirped CLPG.

For the case with an operating wavelength from 1.30 to 1.62 μm, we simulated the PCMs of higher order modes of the chirped CLPG, as shown in Figure 7. The corresponding pitch ranges for different modes are illustrated by the shaded regions. The HE_1,11_ leaky mode is selected to couple with the core mode, and the pitch is from 390 to 395 μm for the operating wavelength range. Similar to the design process of the case with an operating wavelength from 1.50 to 1.62 μm, the coating RI was set to be 1.7, and the grating length was set to be 8 cm. The transmission of the RCPCM and the corresponding loss factor of the chirped CLPG were simulated with these parameters and are depicted by the solid and dashed lines, respectively (Figure 8). A circular polarizer with a bandwidth of over 300 nm was achieved. Nevertheless, a peak appears at the center of the transmission curve, which can be eliminated by expanding the maximum grating pitch to 397 μm. Finally, a flat transmission band is obtained, as shown by the dash-dotted line in Figure 8.

## 4. Conclusions

A broadband circular polarizer based on a coated chirped CLPG was proposed and demonstrated by simulations. The coating RI of the chirped CLPG was chosen to be higher than that of the fiber cladding, enabling the coupling of RCPCM with LCPLM. Because of the leakage of LCPLM, the power coupled to the LCPLM from RCPCM will radiate out of the fiber and cannot be recoupled back to the RCPCM, which is desired for a high extinction ratio. The coupling mechanism between RCPCM and LCPLM was analyzed with complex coupled-mode theory, and the condition under which the RCPCM decays the most quickly was identified. Moreover, the PMTP was used in the design, which resolved the conflict between the operating bandwidth and the grating pitch range of the chirped CLPG and made a large bandwidth with a small grating pitch possible. 

The proposed design scheme efficiently combines the leaky mode coupling and PMTP. Using the design scheme, two broadband circular polarizers with an extinction ratio of over 25 dB were demonstrated for various applications: one with a bandwidth of over 120 nm and a length of 3.5 cm and the other with a bandwidth of over 300 nm and a length of 8 cm. The designed bandwidth matches that of commercial linear polarizers, so we believe that it is large enough for most applications. 

On the other hand, to make full use of the PMTP, higher-order leaky modes are used in the design, which leads to a short twist pitch and increases the requirement for twist pitch control. However, as the twist pitch in our design is still with the same magnitude compared with that of conventional CLPG [29], we consider that the increased fabrication difficulty can be overcome. Our further work will focus on the fabrication and applications of CLPGs. 

To prospect, the propose circular polarizers are based on commercially available Hi-Bi fibers, have an all-fiber structure, and hold considerable potential for applications in optical communication and fiber sensing. The properties of leaky mode coupling and the combination of leaky mode coupling with PMTP will inspire the design of other fiber-based devices.

## Data Availability

Not applicable.
